# Predisposition of Women to Cardiovascular Diseases: A Side-Effect of Increased Glucocorticoid Signaling During the COVID-19 Pandemic?

**DOI:** 10.3389/fgwh.2021.606833

**Published:** 2021-02-16

**Authors:** Hemangini A. Dhaibar, Diana Cruz-Topete

**Affiliations:** Department of Molecular and Cellular Physiology, Center for Cardiovascular Diseases and Sciences, Louisiana State University Health Sciences Center, Shreveport, LA, United States

**Keywords:** COVID-19, pandemic, women, cardiovascular risk, stress, glucocorticoids, heart

## Abstract

The novel coronavirus disease 2019 (COVID-19) pandemic has created a significant health crisis worldwide. To mitigate this disease's spread, “social distancing” and “shelter in place” have been implemented. While these actions have been critical to controlling the pandemic, they have short- and long-term mental health consequences due to increased stress. There is a strong association between mental stress and cardiovascular disease (CVD). Young women (pre-menopausal) are at high risk of developing CV events in response to mental stress compared to age-matched men. The mechanisms underlying women's increased reactivity and response to stress are mostly unknown. The present review summarizes the known physiological consequences of mental stress in women's CV health and the latest molecular findings of the actions of the primary stress hormones, glucocorticoids, on the CV system. The current data suggest a clear link between psychological stress and heart disease, and women have an increased sensitivity to the harmful effects of stress hormone signaling imbalances. Therefore, it is expected that with the given unprecedented levels of stress associated with the COVID-19 pandemic, women's CV health will be significantly compromised. It is critical to widen our understanding of the direct contribution of mental stress to CVD risk in women and to identify biochemical markers with predictive value for CVD in female patients with/without cardiovascular conditions who have experienced significant mental stress during the current pandemic.

## Introduction

An outbreak of a novel coronavirus that started in December 2019 in Wuhan, China, has resulted in a horrifying pandemic (1). Worldwide, the health and economic effects of the coronavirus disease 2019 (COVID-19) have been exacerbated for women, in particular for young-middle aged women, who are struggling to combine their professional and family responsibilities (2–4). Disparities in job security, wages, and social pressure to stay home to care for children and older family members have significantly heightened psychological and physical pressure for women as compared to their male counterparts (5). Moreover, with the deepening pandemic situation, the restricted movement and social isolation measures have led to an exponential increase in gender-based violence (6). Therefore, women are currently suffering from an unprecedented level of psychological and physical stress.

Exposure to acute and chronic mental stress has been associated with an increase in the causation of pathological conditions for both men and women; however, women are more susceptible to the deleterious effects of stress compared to men (7, 8). Depression and anxiety are associated with an increased incidence of obesity, autoimmune disorders, and atherosclerosis in women (9). Clinical studies have highlighted the connection between elevated mental stress and adverse cardiovascular events in women, including myocardial ischemia (MI) and stroke (10–12). Mental stress–induced MI (MSIMI) is twice as common in women under 50 years old than similarly aged men (13). Moreover, among patients with coronary artery diseases (CAD), women, especially younger women, are more likely to develop MSIMI than men, despite less severe obstructive CAD and a relatively similar profile of traditional CAD risk factors (14). Despite these mentioned clinical evidences, the molecular pathways underlying the deleterious effects of stress in women are unknown. In the present review, we summarize the known sex-specific molecular and physiological effects of stress (crosstalk between sex and stress hormones) on the cardiovascular system and discuss the clinical manifestations of mental stress on the female heart. We also review the potential implications of the elevated mental stress associated with the COVID-19 pandemic in context of future cardiovascular risks in women.

## Stress Hormone Signaling and Physiological Effects

Any stimulus, intrinsic or extrinsic, that evokes a biological response can be considered as stress (15). These stress stimulating factors can be environmental, inflammatory, psychological, or physical. Exposure to stress leads to the activation of the hypothalamic-pituitary-adrenal (HPA) axis. The effect of stress on the central nervous system (CNS) was first demonstrated in 1968 when studies by Bruce McEwen showed the effects of adrenal hormones on reconfiguring network connections on the brain (16). McEwen's work provided a direct evidence of the chronic effects of cortisol (primary stress hormones in humans) on mental function regulation and coined the term “allostatic load” as the process by which the body prepares and responds to stress to restore homeostasis. His work demonstrated that chronic exposure to stress lead to major changes in neuronal network connections that triggered a neuroendocrine response associated with multi-organ effects (17). McEwen's pioneer work also indicated that chronic stress exposure contributed to neurodegenerative diseases and that stress had sex-specific effects on the CNS (17). The classic primary endocrine mechanism of a body in response to stress encompasses the production of glucocorticoids.

### Regulation of Glucocorticoid Secretion and Molecular Signaling

**Figure 1:**
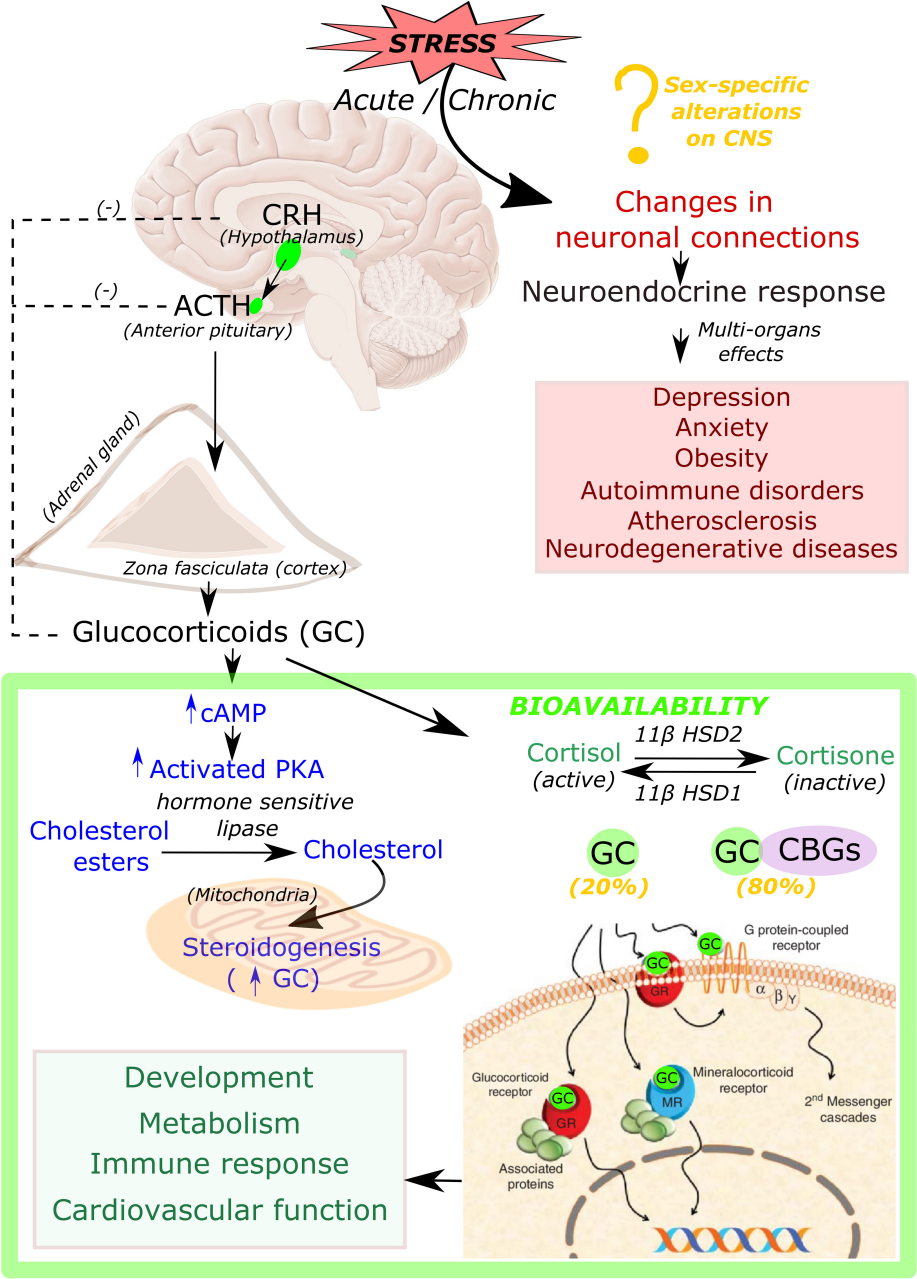
Synthesis, bioavailability and role of glucocorticoid hormone in response to stress. Acute and chronic exposure to stress stimulate hypothalamus to release corticotropin releasing hormone (CRH). CRH then triggers the secretion of the adrenocorticotrophic hormone (ACTH) from the anterior pituitary gland, which binds to its receptors located on the cortex of the adrenal gland that leads to production of intracellular cyclic adenine monophosphate (cAMP). cAMP then activates protein kinase A (PKA). This activated PKA phosphorylate cAMP response element-binding protein (CREB), eventually promotes production of steroidogenic proteins that transport cholesterol into the mitochondria, where glucocorticoids (GC) are synthesized (steroidogenesis). Biologically active form of GC is present in the unbound form (20%), whereas 80% remains in inactive condition bound to corticosteroid-binding globulin (CBGs). Free active GC binds to glucocorticoid receptor (GR) or mineralocorticoid receptor (MR) which leads to further downstream signaling responsible for many physiological processes such as development, metabolism, immune response, and cardiovascular function. Chronic stress also leads to changes in many neuronal connections leading to pathological conditions such as depression, anxiety, obesity, autoimmune disorders, atherosclerosis as well as neurodegenerative diseases.

Glucocorticoids are steroid hormones that are essential for life and are synthesized in the adrenal cortex in response to signals from the hypothalamus (Figure 1). Stress stimulates the paraventricular cells in the hypothalamus to produce the corticotropin-releasing hormone (CRH). CRH is then released into the pituitary portal vein that stimulates corticotrophs in the anterior pituitary gland for the synthesis and release of adrenocorticotropic hormone (ACTH). ACTH then binds to G protein-coupled receptors located on the zona fasciculata and zona reticularis of the adrenal cortex, which then leads to an increase in intracellular cyclic adenosine monophosphate (cAMP) and activation of protein kinase A (PKA). PKA in turn phosphorylates and induces hormone-sensitive lipase to hydrolyze cholesteryl esters into cholesterol (18) as well as activates the steroidogenic acute regulatory protein (StAR) (19–21), which then transports cholesterol into the mitochondria, where glucocorticoids are synthesized in a process known as steroidogenesis.

Chronic production of high levels of cortisol results in Cushing syndrome, also known as hypercortisolism (22), while insufficient amounts of adrenal hormones (cortisol, or cortisol and aldosterone) can lead to Addison's disease (23). Both of these conditions involve the dysfunction of HPA axis signaling and have been linked to immune, metabolic, cardiovascular, and mental conditions such as melancholic depression and chronic anxiety (24, 25). Therefore, tight control of glucocorticoid secretion is critical to maintain homeostasis. Glucocorticoid levels are regulated by a negative feedback loop at the level of the hypothalamus and pituitary gland. Following the hormone secretion, bioavailability of glucocorticoid is regulated by binding to corticosteroid-binding globulins (CBGs). It is estimated that 80% of circulating cortisol is bound to CBGs (26). At target tissues, glucocorticoid availability is further modulated by the action of two enzymes: 11β-hydroxysteroid dehydrogenase type 2 (11βHSD2) which oxidizes cortisol into the inactive metabolite cortisone, whereas 11β-hydroxysteroid dehydrogenase type 1 (11βHSD1) converts cortisone to cortisol (Figure 1). After release from CBGs, free glucocorticoids can diffuse through the cell membrane, and, once inside the cell, glucocorticoids bind their receptor, the glucocorticoid receptor (GR, NR3C1) (Figure 1).

Glucocorticoid receptor (GR, NR3C1) is a member of the nuclear receptor family of ligand-activated transcription factors, which is expressed in almost every cell in the body (27). Binding of glucocorticoids to GR results in the receptor-glucocorticoid complex translocation into the cell nucleus where GR directly (biding to DNA) or indirectly (interaction with other transcription factors) regulate the expression of target genes (28). Glucocorticoids via GR binding can regulate a vast array of genes involved in controlling the development, metabolism, immune response, and the cardiovascular system (29). Endogenous and some synthetic glucocorticoids can also bind to closely related mineralocorticoid receptor (MR, NR3C2), which is not as widely expressed as the GR, but high levels of MR has been observed in cardiovascular tissue (30). The main ligand for MR is aldosterone. However, giving the fact that cortisol circulates at ~100 times higher concentrations than aldosterone, in certain tissues that lack 11βHSD2, glucocorticoids have been found to significantly occupy MR (30). In the context of the cardiovascular system, glucocorticoid activation of GR has been found to be beneficial for the body to restore homeostasis; however, binding to MR has been shown to exacerbate cardiac dysfunction and failure (31). However, no studies have been performed to evaluate the sex-specific effects of glucocorticoids signaling through MR or GR. The structure and function of the GR gene and protein, and mechanisms of gene regulation are discussed in detail in a recent review by Scheschowitsch et al. (32).

### Glucocorticoids and the Cardiovascular System

Glucocorticoids have positive effects on the cardiovascular (CV) system. Treatment with synthetic glucocorticoids can provide beneficial therapeutic effects on conditions such as myocarditis, cardiac conduction defects, as well as vascular conditions such as angina and acute myocardial infarction (33). However, due to the existence of severe side effects in off-target organ systems, the therapeutic use of glucocorticoids is limited.

**Figure 2:**
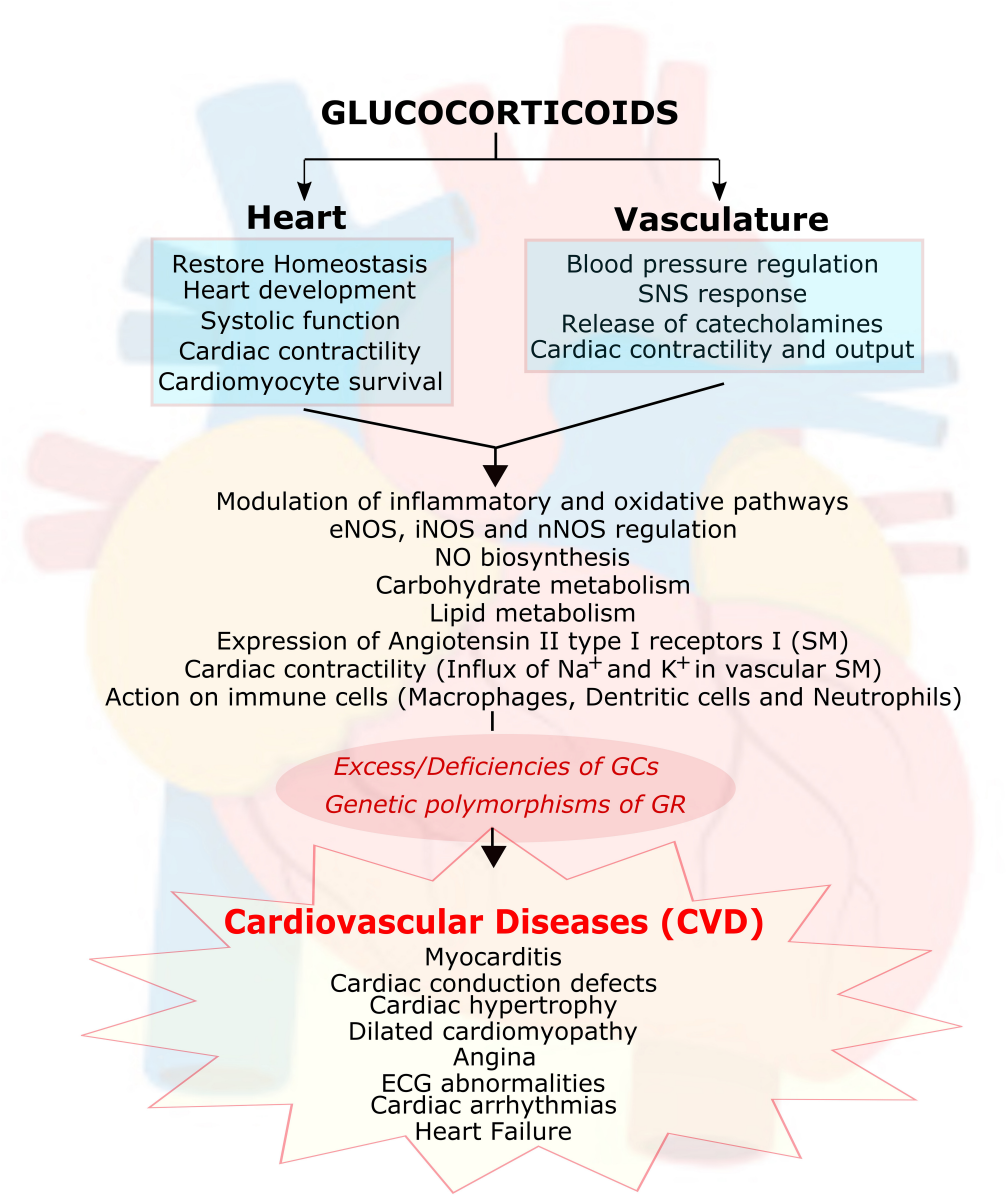
Essential role of glucocorticoids in cardiovascular function. Glucocorticoids (GC) play an essential role in heart and vasculature. It is involved in signaling important functions such as maintaining the cardiac homeostasis, cardiac development, cardiac contractility, cardiac rhythm, modulation of inflammation and oxidative stress, cardiomyocyte survival, carbohydrate and lipid metabolism, inhibiting nitric oxide (NO) biosynthesis, modulating expression of angiotensin II type I receptors on smooth muscle cells (SM) as well as interaction with immune cells (macrophages, dendritic cells and neutrophils). Abnormal function of (GC) due to its excess/deficiencies or due to genetic polymorphism in its receptor leads to many cardiovascular diseases. GC, glucocorticoids; GR, glucocorticoid receptors; eNOS, endothelial nitric oxide synthase; iNOS, inducible nitric oxide synthase; nNOS, neuronal nitric oxide synthase; SNS, sympathetic nervous system; ECG, electrocardiogram.

In normal physiology, both excesses, and deficiencies of glucocorticoids can lead to cardiovascular disease (CVD) (34). Hypertension and cardiomyopathies are commonly found in Cushing Syndrome patients (35, 36). However, hypotension and cardiac dysfunction are regarded as signs of cortisol insufficiency. Polymorphisms of the GR gene are also reported to influence the progress and prognosis of CVD in humans (37–44). The actions of glucocorticoids on the vasculature and the heart are summarized in Figure 2.

In the vascular system, glucocorticoids have been shown to be involved in blood pressure regulation through the modulation of inflammatory and oxidative stress molecular pathways (29, 44–46). In addition, as discussed above, glucocorticoids have been confirmed as vital hormones in the regulation of blood pressure (BP) (36, 47), and there is strong evidence that GR is present in both vascular smooth muscle (VSM) (47) and endothelial cells (48). Also, clinical and animal studies have shown that glucocorticoid signaling is critical in the heart (31, 33, 36, 49–52). Antenatal exposure to glucocorticoids increases the expression of endothelial nitric oxide synthase (eNOS, critical for the production of nitric oxide, which is one of the most important endogenous vasodilators) in the large vessel endothelium, large airway, and small airway epithelium of fetal rat lungs (53, 54). In adult animal models, exogenous glucocorticoid administration leads to hypertension by suppressing nitric oxide synthase III (NOS) and inducible nitric oxide synthase (iNOS) expression (47, 55–57). Glucocorticoid treatment also inhibits nitric oxide (NO) biosynthesis in the endothelium (48). In addition, glucocorticoids increase the expression of angiotensin II type I receptors in smooth muscle cells, and the influx of Na^+^ and Ca^2+^ into vascular smooth muscle affects contractility and therefore leads to alterations in blood pressure (58). Moreover, glucocorticoids are known to exert actions on the vasculature by their effects on immune cells, including on macrophages, dendritic cells, and neutrophils (29, 59–62). It is still controversial whether glucocorticoids' actions on the vasculature are mediated through GR or MR. Future studies are needed to fully elucidate if glucocorticoids can contribute to hypertension via GR or MR.

In the last decade, a number of studies have been focused on understanding the direct effects of glucocorticoid signaling on the heart. Studies have shown that glucocorticoids signaling through GR or MR play a critical role in regulating cardiac function in health and disease (63). In addition, glucocorticoid signaling through GR contributes to heart development. Using mouse models lacking GR in cardiomyocytes and vascular smooth muscle cells indicated that structural, functional, and biochemical maturation of the fetal heart is dependent on intact glucocorticoid signaling (64). Studies on adult mice with cardiomyocyte GR deficiency have also exhibited that an intact glucocorticoid signal is critical for the regulation of systolic function in a post-natal heart. Cardiomyocyte GR deficiency in adult mice leads to early death due to pathological cardiac hypertrophy that progresses to dilated cardiomyopathy and heart failure (50). These effects seem to be associated with the GR regulation of genes involved in cardiac contractility (ryanodine receptors 2, RyR2), cardiomyocyte survival (prostaglandin D2 synthase, Ptgds), and the inhibition of inflammation (lipocalin 2, Lcn 2) (50). MR deficiency does not lead to any major structural or functional abnormalities, and it seems to be protective against myocardial injury (65, 66). GR overexpression in the heart leads to bradycardia and a chronic atrioventricular block in mice (49) but not arrhythmia or premature death. In contrast, MR overexpression and increased signaling in the heart leads to major ECG abnormalities, cardiac arrhythmias, dysregulation in Na^+^ and K^+^ currents, and a high death rate (67). Whether glucocorticoid effects in the heart are mediated via GR or MR is a topic of controversy. However, recent novel studies by Oakley et al. (31) provide direct evidence that glucocorticoid signaling through MR in the absence of GR in cardiomyocytes seems to mediate most of the negative effects of glucocorticoids in the heart. Glucocorticoids signaling via cardiomyocyte MR leads to cardiac pathology, whereas glucocorticoids signaling through GR have been observed to be cardioprotective. Thus, these results suggest that the balance between GR and MR is critical in heart disease. However, it remains to be clarified whether the effects of glucocorticoid signaling on the heart are sexually dimorphic.

### Glucocorticoid Signaling Cross-Talk With Sex Hormones

The sexually dimorphic actions of glucocorticoid regulation of gene expression were observed to contribute to the dimorphic basis of inflammatory disease in a study by Duma et al. (68). In this study, comparison of number of genes involved in inflammatory disorders between sexes revealed that glucocorticoids have more profound anti-inflammatory effects on males as compared to females, suggesting that females have additional factors that may inhibit/alter the response to glucocorticoids (68).

GR exhibits female-biased expression in several preoptic and thalamic nuclei, thus indicating that glucocorticoids have a greater influence on physiology and behavior, mediated by specific neuropeptides more so in females than in males (69). Since the brain plays an important role in governing the stress response, this may contribute to gender differences in CV response to stress. The CV system is susceptible to emotional stress, and young and middle-aged women appear to be especially vulnerable to psychosocial risk factors (11, 13, 70–72). Depression, trauma, and perceived stress are disproportionately common in women as compared to their male counterparts or older patients and can be considered predictors of CV risk (14, 73–75). However, no studies have been performed to investigate whether exposure to severe mental stress for a considerable period of time leads to irreversible gene programming and epigenetic changes that predispose or increase the risk for CV complications, despite going back to a period of “normal” stress levels.

Regarding the sexual dimorphic effects of glucocorticoid on the heart, animal studies have demonstrated that the deletion of GR in cardiomyocytes leads to systolic dysfunction and heart failure in both male and female mice (52). However, this phenotype appears early in males as compared to females and is associated with dysregulation of different cardiac gene networks (52). These differences may arise from the effects of sex hormones on the heart. Ovarian hormone (in particular, estrogen) signaling may be compensating initially for the lack of GR in the heart, whereas androgens may be exacerbating the deleterious effects of GR deficiency in cardiomyocytes (52, 68, 69, 76–78). Future studies are needed to fully elucidate the mechanisms behind the sex differences in the physiological consequences of GR signaling in the heart. However, more work is needed to clarify whether glucocorticoid signaling in heart results from GR cross-talk with androgen receptors (AR) or estrogen receptor (ER) signaling and whether if these interactions play a role in male and female differential sensitivity to the effects of exposure to higher stress levels as it relates to cardiovascular and heart disease. Moreover, studies are needed to further define the role of MR in glucocorticoids' sex-specific effects on the heart.

In addition, chronic stress has been shown to increase the risk of hypertension for both men and women (79, 80). Most studies have associated stress and hypertension with the stimulation of the sympathetic nervous system response, in which the release of catecholamines leads to increased heart rate, cardiac output, and altered blood pressure (80). However, future studies need to focus on investigating the direct contribution of glucocorticoid release in response to stress in blood pressure regulation, with special emphasis on characterizing the gender-specific effects of chronic stress and pathological hypertension.

In the next section, we briefly discuss how trauma-related mental health disorders during the COVID-19 pandemic might alter glucocorticoid signaling in the female heart, and the potential CV side-effects of the increased activation of GR signaling associated with the COVID-19 pandemic for women.

## The Price of the COVID-19 Pandemic Associated Stress on Women's CV Health

The COVID-19 pandemic has led to unprecedented levels of mental and emotional stress (81). The uncertainty due to the fear of infection, economic losses, and isolation due to quarantining has triggered a substantial decline in mental health for both men and women. However, women's mental health appears to be disproportionally affected. Emerging data show that women are suffering more than men from the pandemic-associated stressors, and that there is a higher self-reported symptoms of anxiety, depression, post-traumatic stress disorder, and poor psychological well-being in them (82–85). Moreover, since women are already at a higher risk for depressive and anxiety disorders, the current environmental stress has intensified the severity of these disorders for women (86).

There is a strong association between psychological stress and cardiovascular disease (70, 87–90). Exposure to stressors such as natural disasters has demonstrated an increase in cardiovascular risk associated with prolonged emotional trauma due to human and economic losses and changes in the daily routine. Studies show that sudden changes in heart rate and increases in blood pressure are common in populations that have experienced an earthquake and are facing uncertainty (90, 91). Moreover, a dramatic increase in pulmonary embolism and myocardial infarction (MI) has been observed in the wake of an earthquake (91). Similarly, other natural disasters, such as hurricanes, floods and tsunamis, that disrupt the fully functioning lives of the victims and cause loss for individuals, families and communities have highlighted the association between CVD risk and mental stress (92, 93). A recent study also revealed that the number of trauma-related mental health disorders has increased significantly during COVID-19 quarantine (83, 94). Therefore, a substantial increase in mental health conditions and associated sequelae is expected to be a consequence of this pandemic worldwide. Given the link between mental stress and CVD risk, it is critical to investigate the biological pathways underlying the stress response and the CV system to identify patients at risk (prevention) and to discover novel therapeutic targets.

Traditionally, it has been assumed that premenopausal women have a lower cardiac risk than men (95). This decreased risk has been attributed to estrogen, which has anti-atherosclerotic effects (96–98). Data from the Framingham Heart Study suggested a strong association between low estrogen levels (menopause) and increased cardiovascular risk in women (99). However, while some studies show that low estrogen dose therapy has been shown to be beneficial for cardiovascular health in post-menopausal women (100), the data remain controversial regarding whether long-term estrogen therapy improves cardiovascular outcomes for women (101). Moreover, recent clinical evidence has also shown that although there has been a decrease in heart disease mortality for both men and women over 65 years of age in the last three decades, the incidence of cardiovascular events has significantly increased among premenopausal women (102). These results suggest that additional risk factors have a differential impact on women's cardiovascular health compared to men.

**Figure 3:**
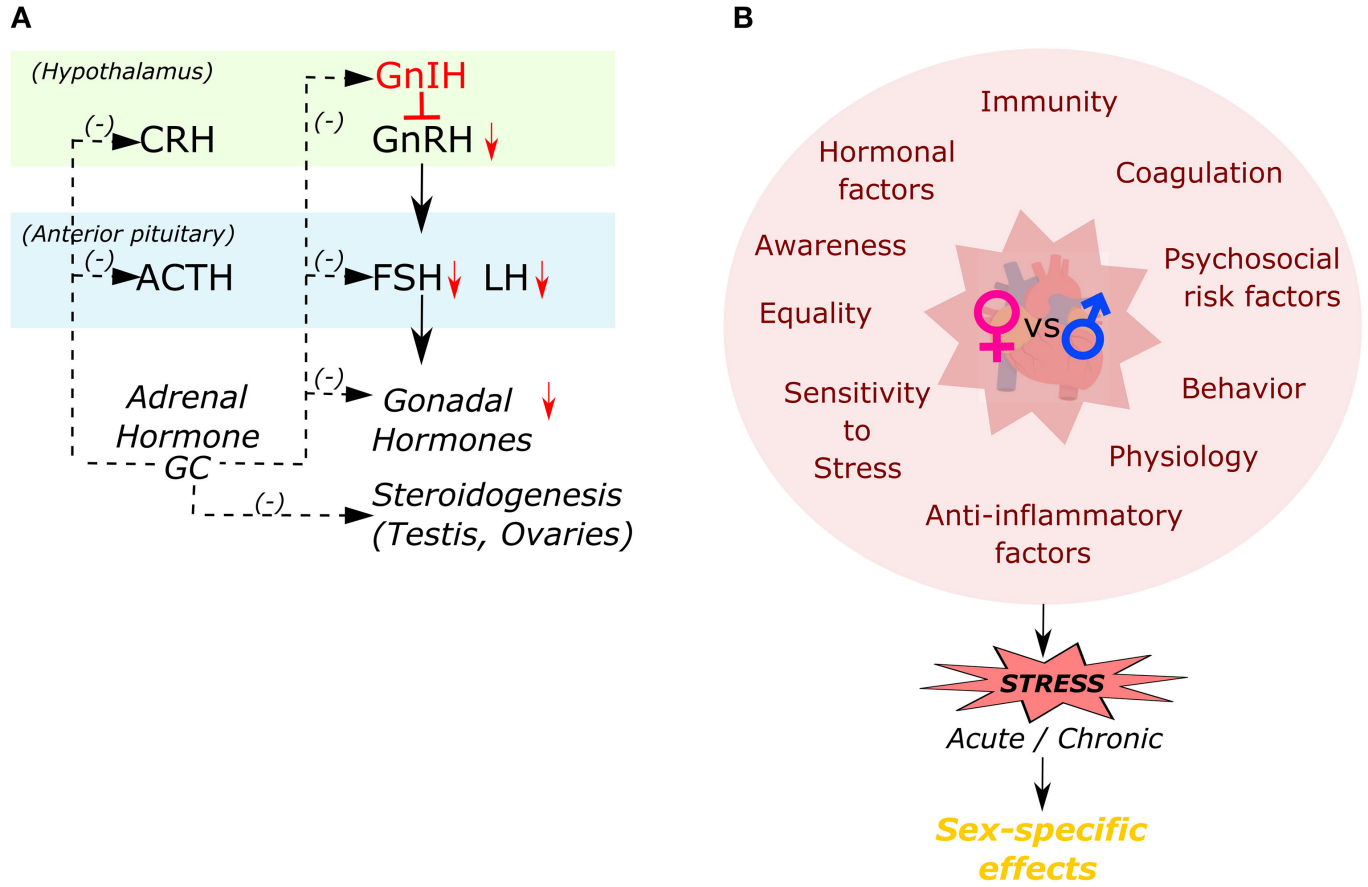
Inter-relationship of different factors responsible in causation of sex-specific effects to stress. **(A)** Interactions among the hypothalamo-pituitary-adrenal (HPA) and hypothalamo-pituitary-gonal (HPA) axes by glucocorticoid hormone (GC). **(B)** Different factors influencing female's cardiac health compared to males in developing a sex-specific effect in response to stress.

Women differ from men in a multitude of ways (Figure 3), including genetic differences in immunity (103, 104), coagulation (105, 106), and hormonal factors (107), all of which can influence the risk for CVD and related outcomes. Many studies have highlighted sex differences in delayed hospital arrival and lack of sufficient awareness of women in the context of CVD (108, 109). Along with these factors that has been associated with increased mortality for women, abnormal levels of glucocorticoids have also been known to increase CV risk for women (7, 11, 75, 110–112). However, surprisingly, very little has been explored about the direct role of glucocorticoid signaling on the female heart.

## Conclusions and Future Perspectives

The COVID-19 pandemic has exponentially raised anxiety and depression in vulnerable populations due to economic and social pressure, uncertainty, isolation, and feelings of immobility/constraint due to social distancing measures. Young and middle-aged women are among the most affected due to the lack of balance between demanding job schedules and family responsibilities.

The mechanisms responsible for the sex-specific effects of stress hormones on the CV system are still unclear. Women have an increased vascular reactivity to glucocorticoids, which may account for their increased risk of mental-stress-induced ischemia (73, 113). However, the molecular pathways underlying this reactivity are unknown. A potential mechanism for the sex-specific effects of stress is the crosstalk between glucocorticoids and sex-hormones signaling. A better understanding of such interactions will open up new potential avenues for risk assessment and prevention for women. It will be particularly be important to study whether exposure to chronic mental stress for a period of time leads to gene reprogramming that may predispose women to CV complications, exacerbate the effects of additional comorbidities, and negatively impact the aging process. Assessment of mental health status, in addition to traditional risk factors, has become more important than ever. There is a clear connection between psychological stress and heart disease and understanding this connection will aid in preventing and improving cardiovascular outcomes for the general population and women.

## Author Contributions

All authors contributed to writing the manuscript, critically revised the work, and approved the final version.

## Conflict of Interest

The authors declare that the research was conducted in the absence of any commercial or financial relationships that could be construed as a potential conflict of interest.
